# Evaluation of Nanodiamond-in-Oil Emulsion with Snake Venom to Enhance Potent Antibody Induction in Mice and Rabbits

**DOI:** 10.3390/nano15191518

**Published:** 2025-10-04

**Authors:** Min-Han Lin, Long-Jyun Su, Hsin-Hung Lin, Liang-Yu Chen, Asmaul Husna, Wang-Chou Sung

**Affiliations:** 1 National Institute of Infectious Diseases and Vaccinology, National Health Research Institutes, Zhunan 35053, Taiwan; hamlyn@nhri.edu.tw; 2 LuminX Biotech Co., Ltd., Taipei 11503, Taiwan; harrysu@luminxbiotech.com (L.-J.S.); hhlin@luminxbiotech.com (H.-H.L.); amberchen@luminxbiotech.com (L.-Y.C.); 3 College of Life Science, National Tsing Hua University, Hsinchu 30013, Taiwan; asmaulhusna.dpp@sau.ac.bd

**Keywords:** nanodiamonds, cobra venom, lesion effect, nanodiamond-in-oil formulation, antivenom

## Abstract

Nanodiamonds (NDs) are an innovative material in biomedical applications based on their excellent biocompatibility, nanoscale dimensions, and high surface area. In this study, we evaluated the potential of ND-in-oil emulsion to induce potent antibody responses in animals immunized with cobra venom. NDs demonstrated the capacity to bind complex venom proteins as stable conjugates, well dispersed in aqueous solution. Immunization of mice with cobra venom incorporated with ND-in-oil emulsion adjuvant (ND/venom) elicited strong venom-specific antibody responses with titers comparable to those induced by venom formulation with conventional Freund’s adjuvants (FA/venom). IgG subclass analysis revealed that ND- and FA-based formulations induced a Th2-biased immune response in mice. Moreover, antibodies elicited by ND/venom or FA/venom immunization specifically recognized the epitopes of the lethal component of short-chain neurotoxin and conferred full protection against lethal cobra venom challenge (3LD_50_). Further, ND/venom hyperimmunization was capable of inducing high levels of neutralizing antibodies in larger animals, rabbits, highlighting the potential for antivenom manufacturing. Notably, there were no obvious lesions at the injection sites of animals that received ND/venom, in contrast to those that received FA/venom. These findings indicated NDs as an effective and safe additive in venom formulation for antivenom production.

## 1. Introduction

Snakebite envenoming remains a significant health threat in tropical and subtropical areas. There are estimated to be 5 million snakebite cases every year in the world, resulting in 100,000 deaths and numerous permanent disabilities [1,2]. Administration of antivenom is the main treatment recommended by the World Health Organization for snakebite victims. Antivenoms contain polyclonal antibodies purified from the plasma or serum of large animals, like horses or sheep, that received hyperimmunization with venom formulations [3]. However, the global supply of antivenoms remains critically limited, mainly due to the manufacturing capacity, complex production processes, and the variability of snake venoms [4]. To reduce morbidity and mortality associated with snakebite envenoming, efforts have been made to investigate strategies to enhance the availability and quality of antivenoms [5].

Hyperimmunization is a strategy to enhance antibody induction in experimental animals by the repeated injections of adjuvanted venom formulations. Traditionally, both complete and incomplete Freund’s adjuvants have been extensively employed to formulate snake venom as an immunogen for antivenom production. This approach has been demonstrated to effectively induce robust antibody responses in animals. Complete Freund’s adjuvant (CFA), containing heated-killed *Mycobacterium*, is generally used in prime immunization, which is reported to recruit the antigen-presenting cells (APCs) and promote innate activation pathways to stimulate immunity. Incomplete type Freund’s adjuvant (IFA), which lacks the bacterial component, functions to boost antibody responses through the depot formation at the injection sites. Despite their immunostimulatory efficacy, CFA has been reported to cause lesions and tissue necrosis in immunized animals [6], potentially reducing the quality and consistency of antivenom batches over time. To address the concerns, many commercial adjuvants, like mineral salt adjuvant [7], bentonite [8], CpG oligodeoxynucleotides [9], Emulsigen-D [10], and liposome [11] have been investigated; however, they offer improved safety and reduced lesions, while their immunogenicity and toxic effects in other animals remain unclear.

Nanodiamonds (NDs) are carbon-based nanomaterials with excellent biocompatibility and chemical inertness, which have been widely utilized for cellular imaging [12], drug delivery [13], and biosensing probes [14]. Owing to their high surface area and tunable surface chemistry, NDs have also been investigated as antigen carriers to enhance antigen delivery and presentation to immune cells [15]. Previously, a study by Pham et al. demonstrated that protein-conjugated ND-in-oil emulsion is efficiently taken up by macrophages and bone-marrow-derived cells, enhancing greater antibody responses compared to protein alone [16]. Regarding biosafety, Lin et al. show that NDs are non-toxic to mice and can be excreted from the body [17]. Given the high safety profile and ability to enhance effective antibody responses, NDs are currently being explored for applications in novel vaccine and therapeutics development.

*Naja atra*, commonly known as the Taiwan cobra, is a venomous snake species endemic in tropical and subtropical regions. In Taiwan, cobra bites account for nearly 20% of snake envenoming cases annually [18]. Previously, our proteomic analysis of Taiwan cobra venom identified eight protein families, each with diverse physical and biological properties that act synergistically to induce severe clinical manifestations in envenomed victims [19]. Of them, the short-chain neurotoxin (sNTX) is classified as the medically important toxin based on its abundance and potent neurotoxicity, which functions to block muscular-type nicotinic acetylcholine receptors (nAChRs) [20,21], thereby contributing significantly to the lethality of cobra venom. Compared to other venom components, sNTX is a low molecular weight protein with inherently low immunogenicity, making it a challenging target for effective antibody generation in immunized animals. Approaches to enhance the induction of antibodies against key venom toxins have been reported previously; these include the use of purified toxins as immunogen [22,23]; a virus-like particle displaying linear toxin-derived peptides [24]; and a prime-boost immunization protocol combining DNA plasmid encoding toxin as the priming agent, with the crude venom for boosting [25]. However, the production purification of toxin targets or recombinant antigens often require extensive procedures, which is ultimately unfeasible in industrial antivenom manufacturing.

In the aspect of immunogenicity enhancement, NDs function as an effective protein carrier platform to improve the recognition of immune cells and promote antibody induction. In this study, cobra venom was formulated with ND-in-oil emulsion (ND/venom) or conventional Freund’s adjuvants (FA/venom) to compare their immunogenicity in eliciting potent antibody responses in mice and rabbits. Various amounts of cobra venom were mixed with the NDs to assess the binding capacity with the complex protein mixture. Further, dynamic light scattering (DLS) was conducted to profile the size distribution of the resulting particles in deionized water. To understand the immunity induced by ND/venom, enzyme-linked immunosorbent assays (ELISA) were conducted to analyze the titers of specific antibodies and IgG subclasses in sera from the immunized mice. At last, ND/venom and FA/venom formulations were used to immunize rabbits through a hyperimmunization protocol to assess the potential for antivenom production.

## 2. Materials and Methods

### 2.1. Venom and Material

The *N. atra* venom (indicated as cobra venom in the following article) was acquired from a local snake farm in Taiwan. The venom was lyophilized and stored at −20 °C in a refrigerator. The venom solutions were freshly prepared for experimental use.

### 2.2. Production of NDs

The generation of NDs follows the procedures developed in a previous investigation [16]. Briefly, diamond powders were first oxidized in air at 490 °C for 2 h to remove graphitic residues on the surface of the diamond powder (Diamond Innovations, Columbus, OH, USA). Following, the powders were oxidized with a strong oxidative mixture (3 volumes of H_2_SO_4_: 1 volume of HNO_3_) in a microwave reactor (Discover SP, CEM, Matthews, NC, USA) at a heating temperature of 100 °C for 3 h in a chemical fume-hood. The acquired NDs were weighted and suspended in DI water with a 10 mg/mL weight concentration.

### 2.3. Binding Capacity of ND with Cobra Venom

Firstly, cobra venom was detoxified by mixing with glutaraldehyde (0.25%, *v*/*v*) at 37 °C for 30 min, following a previously established protocol [23]. Different amounts of 20, 40, 80, and 160 μg of detoxified venom (10 mg/mL in deionized water) were mixed with a fixed amount of NDs (50 μL, 10 mg/mL) at various weight ratios in solution with a final volume of 100 μL. The mixtures were sonicated for 5 min in a water bath, followed by centrifuging at 17,000× *g* for 10 min (Pico 17, Thermo Scientific, Waltham, MA, USA). The resulting pellets were resuspended in DI water and then analyzed by gel electrophoresis using 4–12% Bis-Tris NuPAGE (Thermo Fisher Scientific, Waltham, MA, USA), followed by Coomassie blue staining. The supernatants were collected and subjected to SDS-PAGE analysis as well. The amount of unbound proteins in supernatant was quantified by a BCA kit (Thermo Scientific Pierce™, Waltham, MA, USA) to determine the binding capacity of NDs for venom proteins by calculating the difference between the initial and unbound venom protein concentrations.

### 2.4. Animal Immunization

Institute of Cancer Research mice (ICR, body weight 19–22 g) were purchased from BioLASCO Co., Ltd. (Taipei, Taiwan) or the Animal Health Research Institute. Animals were housed in the National Health Research Institutes (NHRI) animal center following an approved protocol (NHRI-IACUC-111075-A-S03). Formulation of venom with ND-in-oil emulsion (ND/venom) was prepared following the protocol developed in another investigation [16] with slight modification. Briefly, designed amounts of cobra venom (1 mg/mL in deionized water) were mixed with an equal volume of nanodiamond suspension (10 mg/mL in DI water). After the incubation at room temperature for 30 min, the venom-conjugated NDs were mixed with 65 μL of IFA (Sigma-Aldrich, St. Louis, MO, USA) and then adjusted to 200 μL with water. After vigorous vortexing, the resulting venom emulsion was subcutaneously (s.c.) injected into mice. For Freund’s adjuvant group, the first dose consisted of the venom formulated with CFA (Sigma-Aldrich), and the subsequent booster doses included IFA. Groups of mice (n = 4/group) were immunized with three doses of adjuvanted venom immunogen with a 2-week interval. Two weeks after final immunization, mice were bled to collect the sera and then stored at −20 °C for further analysis.

For rabbit hyperimmunization, the immunization regimen followed the protocol developed by H. Clement [26] with slight modification. Briefly, groups of New Zealand White rabbits (n = 2/group) immunized (s.c.) with ND/venom or FA/venom immunogens were processed in the LTK BioLaboratories (Taoyuan, Taiwan) following an approved protocol (LTK112Rb006). The hyperimmunization schedule consists of five doses with a two-week interval. The amount of cobra venom in each dose was gradually increased from 50 to 200 μg to stimulate antibody induction in rabbits. The immunization with Freud’s adjuvant formulations was given in the same way shown above. Serum samples were collected from the rabbits before each immunization and two weeks after the final immunization. Rabbit sera were stored at –20 °C for subsequent analysis.

### 2.5. ELISA Analysis

Analysis of specific antibody titers in animal sera was performed by ELISA assay using the protocol developed in a previous study [27]. Briefly, the plates were coated with 100 mL of sNTX or crude venom at a final concentration of 0.5 mg/mL in 75 mM sodium carbonate buffer (pH 9.6) at 4 °C overnight. After washing with PBS containing 0.05% Tween-20 (PBST), wells were blocked with 5% skim milk in PBS for 2 h at room temperature. The animal immune sera were decomplemented at 56 °C for 30 min, and then serially diluted (2-fold) in PBS buffer containing 1% bovine serum albumin. The diluted serum samples were added to a 96-well ELISA plate (Corning, Corning, NY, USA) and incubated at room temperature for 2 h. Following, the plates were washed four times with PBS containing 0.05% (*v*/*v*) Tween 20 (PBST). Horseradish peroxidase (HRP)–conjugated goat anti-mouse IgG (GeneTex, Irvine, CA, USA), IgG1 (Bio-Rad, Irvine, CA, USA), and IgG2a (Bio-Rad) were used as secondary antibodies for mouse serum antibody titer measurements. HRP-conjugated goat anti-rabbit IgG (Jackson Lab, Bar Harbor, ME, USA) was used to measure rabbit serum antibody titers. Next, 50 μL of 3,3′,5,5′-tetramethylbenzidine (TMB; KPL, Gaithersburg, MD, USA) was added for color development, followed by termination with 50 μL of 2 N sulfuric acid (H_2_SO_4_), and absorbance at 450 nm was measured on a microplate reader (Sunrise, Tecan, Zurich, Switzerland). Antibody titers were expressed as reciprocal log_10_ titers of serum dilutions corresponding to an optical density (O.D.) > 0.3 at 450 nm. All measurements were performed in duplicate, and the results are shown as the mean ± standard deviation (S.D.).

### 2.6. Peptide-Based ELISA

Overlapping 15-mer synthetic peptides corresponding to the mature sequence of sNTX (accession: P60770) were acquired from the core facility of the NHRI Bioproduction Plant (Table S1). An ELISA assay was conducted to measure serum reactivity against the sNTX-derived peptide fragments as previously described [27]. Briefly, the plates were coated with 100 mL of synthetic peptides at a final concentration of 1 mg/mL in 75 mM sodium carbonate buffer (pH 9.6) at 4 °C overnight. After washing with PBS containing 0.05% Tween-20 (PBST), wells were blocked with 5% skim milk in PBS for 2 h at room temperature. Animal sera were diluted to a concentration of 1:100 and incubated for 90 min at room temperature. The plates were then washed, and all subsequent steps were the same as described above.

### 2.7. Challenge Assay

The assay was conducted in immunized mice to assess the protective efficacy of venom immunizations against cobra venom lethality, following a previously established protocol [23]. Cobra venom with a median lethal dose (LD_50_) of 0.67 μg/g (venom/mouse weight ratio, intraperitoneal, [23]) for ICR mice was dissolved in DI water to prepare the challenge solution. A highly lethal dose, equivalent to 3-fold that of LD_50_ (3LD_50_), was administered intraperitoneally (i.p.) to the experimental mice. Mice were monitored for 48 h post-challenge, and survival rates were recorded to determine the level of protection by each venom formulation.

### 2.8. Neutralization Assay

An in vivo neutralization assay was conducted to evaluate the potency of rabbit hyperimmune sera against the lethality of cobra venom, following the protocol developed in our previous study [23] with slight modifications. Initially, the rabbit antiserum antibodies were purified by the protein G affinity column (5 mL, MabSelect, Prism A, GE Healthcare, Chicago, IL, USA) according to the manufacturer’s instructions. The purified rabbit antibodies were dialyzed to PBS buffer and adjusted to a stock concentration of 40 mg/mL. For the neutralization assay, a fixed amount of cobra venom (40.32 μg, 3LD_50_) was incubated with the varied amounts of purified rabbit antibodies in a final volume of 400 μL. The mixture was incubated at 37 °C for 30 min, then injected (i.p.) into ICR mice (n = 6/rabbit antibody dose group). The mice were monitored for 48 h, and survival rates were recorded to assess the neutralizing potency. The media effective dose (ED_50_) was defined as the amount of rabbit antibodies (mg) required to protect half of the mice from death following exposure to the lethal dose of cobra venom (3LD_50_). The neutralization potency (P) was defined as the minimum amount of antibody needed to completely neutralize the lethal venom dose.

### 2.9. Statistical Analysis

Statistical analysis of the neutralizing potencies of antivenom was performed by one-way analysis of variance, followed by Bonferroni’s multiple comparisons test. A one-way ANOVA with Tukey’s post hoc test was carried out to compare the differences in antibody titers. Results with *p*-values < 0.05 were considered significant. All statistical analyses were performed using Prism software (Version 8.4.3, GraphPad, San Diego, CA, USA).

## 3. Results

### 3.1. Preparation and Characterization of Venom-ND Conjugate

**Figure 1:**
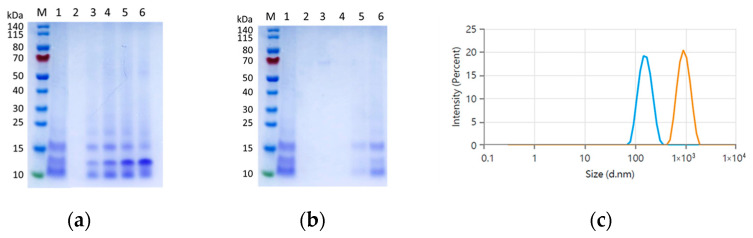
Characterization of bare NDs and the mixture of NDs with venom proteins. SDS-PAGE analysis on (**a**) the precipitates and (**b**) the supernatants from the mixtures of NDs with different amounts of venom after centrifugation. M indicates protein marker. Lane 1 represents the cobra venom. Lanes 2–6 represent the precipitates from the mixtures of NDs mixing with 0, 20, 40, 80, or 160 μg of cobra venoms after sonication and centrifugation. (**c**) Overlay of size distribution of bare NDs (Blue) and ND/venom mixture (Orange) in DI water.

Initially, various amounts of cobra venom (20, 40, 80, and 160 μg, weight) were mixed with a fixed amount of bare NDs (500 μg) and incubated at room temperature for 30 min. After sonication and centrifugation, the supernatants and pellets were collected and analyzed by SDS-PAGE, as shown in Figure 1a. PAGE results revealed that the pellets exhibited clear protein band profiles similar to those of crude venom, demonstrating the capability of NDs to incorporate the diverse protein components of cobra venom. The intensity of protein bands from the pellet samples increased with the weight ratio, and plateaued at samples with a mixing amount of venom over 80 μg. Conversely, protein bands began to appear in the supernatants when the mixing amount of venom exceeded 80 μg (Figure 1b), suggesting the binding capacity of NDs was saturated at this ratio.

Further, the pellet with saturated protein binding was resuspended in DI water to characterize the dispersion status in the solution phase. DLS measurements showed that bare NDs displayed an average diameter of 164 nm in DI water, which increased to a mean value of 948 nm after binding with cobra venom (Figure 1c). The presence of a unimodal size distribution supported that venom proteins could be stabilized on the ND surface, and the particles dispersed well in solution without precipitation. Overall, our results demonstrated the potential of NDs as an efficient carrier with high binding capacity for complex venom protein mixtures.

### 3.2. Induction of Specific Antibody Responses by ND/Venom Immunization in Mice

**Figure 2:**
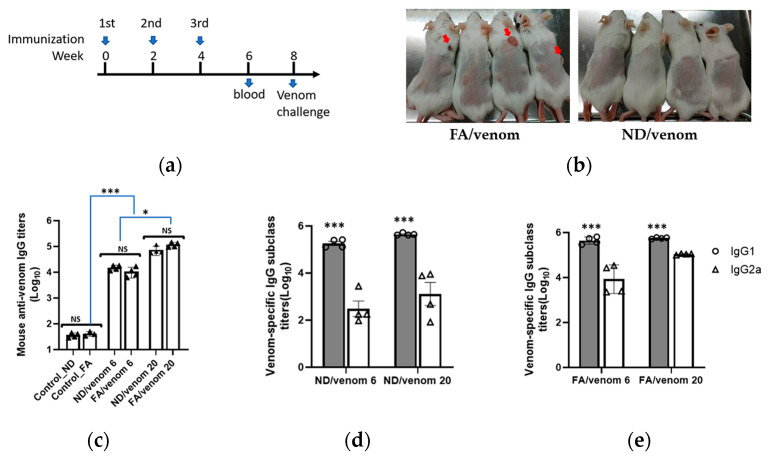
Immunogenicity evaluation of venom formulations with NDs or FAs. (**a**) Schematic representation of the immunization regimen. Mice were immunized at weeks 0, 2, and 4 with the prepared dosages of ND/venom or FA/venom immunogens. Black triangle represent individual data point for each mouse. (**b**) Images of mouse skin after injecting with either FA/venom or ND/venom immunogen. The red arrow indicates the lesion area. The mouse sera at week 6 were collected to measure the titers of (**c**) total IgG antibodies, (**d**) IgG1, and (**e**) IgG2a by ELISA precoated with cobra venom. Data are presented as mean ± SEM. Statistical significance was determined by one-way ANOVA with Tukey’s post hoc test (NS, non-significant, * *p* < 0.05, *** *p* < 0.001).

In the immunization experiment, the NDs incorporated with 20 μg (ND/venom 20) or 6 μg (ND/venom 6) of cobra venom were emulsified with oil adjuvant following the protocol as described in the experimental section. For comparative study, the same dosages of cobra venom formulated with the complete and incomplete Freund’s adjuvant (FA) were prepared for mice immunization. Figure 2a shows a three-dose immunization regimen with a 2-week interval, and mice immunized with the FA or ND alone were taken as controls in the study. Notably, local skin lesions occasionally occurred at the injection sites of mice that received FA-based formulations, while no such adverse effects were found in the ND/venom groups (Figure 2b). Two weeks after final immunization, ELISA results revealed the ND/venom 6 immunization raised the venom-specific antibody response with a titer of 4.2 log_10_ on average, which was comparable to that from the FA/venom 6 group (3.9 log_10_, Figure 2c). For high-dosage immunization, a more than five-fold enhancement of the venom-specific antibody titer was detected in both ND/venom 20 (4.5 log_10_) and FA/venom 20 (4.9 log_10_) groups (Figure 2c). In contrast, no obvious antibody responses were observed in controls.

Further, we analyzed the venom-specific IgG subclasses in mouse sera (week 6). For mouse species, IgG1 and IgG2a are antibody subclasses associated with Th2 and Th1 immunity, respectively [28]. Results revealed that both ND/venom and FA/venom immunizations induced significantly higher levels of IgG1 antibody responses as compared to the IgG2a titers in mouse sera. The IgG1/IgG2 ratios for ND/venom groups were over the value of 2, suggesting the induction of a Th2-biased immune response. For FA/venom groups, the IgG1/IgG2a ratios were close to 1, indicative of balanced Th1/Th2 immune responses (Figure 2d,e). Overall, our study results demonstrated that immunization with ND/venom immunization would effectively induce similar levels of antibody responses in mice as compared to that of FA/venom immunogen.

### 3.3. ND/Venom Immunization Induces Neutralizing Antibody Responses in Mice

**Figure 3:**
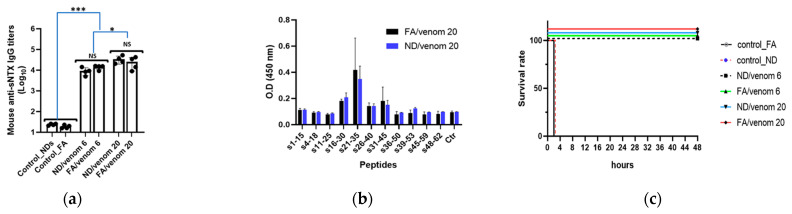
Analysis of specific antibody responses and the protection efficacy induced by cobra venom formulations. (**a**) Endpoint titers of anti-sNTX antibodies in mouse sera collected 2 weeks after final immunization by ELISA. Black dot represent individual data point for each mouse. (**b**) Measurement of mouse sera reactivity to sNTX-derived peptides. The peptide number indicates the amino acid locations of sNTX. A plate well without coating peptide was taken as a blank control (Ctr) in the assay. (**c**) Survival rate of immunized mice for 48 h following the injection of a lethal dosage (3LD_50_) of cobra venom. Data are presented as mean ± SEM. Statistical significance was determined by one-way ANOVA with Tukey’s post hoc test (NS, non-significant, * *p* < 0.05, *** *p* < 0.001).

Accordingly, we analyzed the reactivity of mouse immune serum against the medically important toxin of sNTX purified from cobra venom. ELISA results revealed that ND/venom immunizations can elicit anti-sNTX antibodies in mice, with the titers compared to those by FA/venom, as shown in Figure 3a. Following, the mouse sera from high dosage groups, which have higher antibody titers, were selected to profile the specificity of antibodies by ELISA assay coated with sNTX-derived peptides. Here, a total of 11 peptides (Table S1) with an overlapping sequence of sNTX were applied to profile the specificity of serum antibodies. Results revealed that the mouse sera raised by ND/venom and FA/venom showed reactivity to the peptides between amino acids 16–45, which are located at central loop II of sNTX (Figure 3b).

Subsequently, all immunized mice were challenged (i.p.) with a high dose (3LD_50_) of cobra venom to assess the protective potency of antibody responses induced by either immunogen. After 48 h post-venom injection, all immunized mice except the control groups survived (Figure 3c). Overall, our study results demonstrated that ND/venom immunization could induce specific antibodies against sNTX and sNTX-derived peptide fragments, and the induced antibodies showed the capability to protect mice from the lethal toxicity of cobra crude venom.

### 3.4. ND/Venom Immunization Induces Neutralizing Antibody Responses in Rabbits

**Figure 4:**
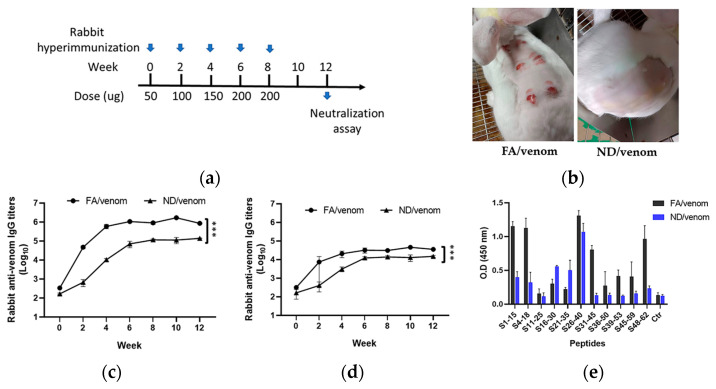
Analysis of specific antibody responses in rabbits following hyperimmunization. (**a**) New Zealand rabbits were immunized with cobra venom adjuvanted with NDs or FA in a five-dose hyperimmunization regimen at a 2-week interval. (**b**) Representative images of injection sites showing skin conditions post immunization with FA/venom (left) and ND/venom (right). Serum antibody titers were determined by ELISA against (**c**) cobra venom and (**d**) sNTX. (**e**) Measurement of rabbit sera reactivity to sNTX-derived peptides. The peptide number indicates the amino acid residues of sNTX. A plate well without coating peptide was taken as a blank control (Ctr) in the assay. Data are presented as mean ± SEM. Statistical significance was determined by one-way ANOVA with Tukey’s post hoc test (*** *p* < 0.001).

Based on the above results, we performed the hyperimmunization with ND/venom in rabbits, a larger animal model, to assess the potential of the immunogen before developing horse antivenom. Figure 4a shows a five-dose immunization regimen for producing the rabbit hyperimmune serum, with a parallel group receiving FA/venom for comparative study. Remarkably, immunization with FA/venom (s.c.) developed lesions at the injection sites, while no adverse signs were observed from the ND/venom group (Figure 4b). Serological analysis demonstrated that FA/venom rapidly elicited the venom-specific antibody responses after the first dose, with titers reaching 5.8 log_10_ by week 4 and maintained at this level until the end of the experiment. The ND/venom group also generated robust antibody responses, reaching 5.0 log_10_ by week 8 and plateauing thereafter. A similar trend was observed in the detection of specific anti-sNTX antibody responses, whereas the FA/venom group reached 4.5 log_10_ compared to 4.0 log_10_ in the ND/venom group (Figure 4c,d). In peptide-based ELISA, results revealed that rabbit hyperimmune sera raised by FA/venom showed a broad reactivity to multiple peptide fragments of sNTX (Figure 4e). The first region was located in the N-terminal portion between amino acids 1–18, the second portion was located in the central loop II between amino acids 16–45, and the third portion was located in the C-terminal portion between amino acids 48–62. In contact, ND/venom hyperimmunization raised the antibodies primarily targeting the amino acids 16–40 at central loop II of sNTX.

To further evaluate the potency of the induced antibodies, we simulated a standard antivenom testing workflow by purifying the immunoglobulins and assessing their venom-neutralizing activity. In the neutralization assay, a series of mixtures containing a high dose of cobra venom (3LD_50_) premixed with the serially diluted rabbit antibodies was injected (i.p.) into mice. Results revealed that the potency (ED_50_)of rabbit antibodies raised by ND/venom hyperimmunization was nearly twofold higher than that of the FA/venom group, as shown in Table 1. One limitation of this study is the number of animals, which may reduce the ability to assess the immunogenic effects of the adjuvant. A higher experimental animal number could potentially provide clearer insights. Overall, our study results demonstrated that hyperimmunization with ND/venom could elicit higher levels of neutralizing antibodies in rabbits without causing lesions, highlighting its potential for antivenom production.

## 4. Discussion

In this study, we demonstrated the potential of ND-in-oil as an effective emulation adjuvant for cobra venom immunization to elicit neutralizing antibodies in mice and rabbits without causing obvious lesions. Specifically, the optimal conjugation conditions between cobra venom and NDs were assessed by SDS-PAGE and BCA assay. Our results showed that efficient binding of venom proteins to NDs can be achieved through directly mixing DI water. The binding capacity of snake venom on the surface of NDs was saturated at a weight ratio of 80 μg venom to 500 μg NDs. Further, SDS-PAGE results revealed that cobra venom is predominantly composed of low molecular weight proteins (Figure 1a, lane 1), which comprise lethal and toxic components from the 3FTXs family and PLA2s [20]. These proteins possess a range of isoelectric points and hydrophobicity, which may facilitate their attachments to the surface of NDs through hydrophobic or electrostatic interactions [17]. Moreover, the venom-conjugated NDs exhibited a uniform particle size, suggesting the incorporation of venom proteins would not affect the dispersion of nanoparticles in the solution (Figure 1c). These characteristics indicate that NDs serve as a suitable carrier platform to stabilize complex venom proteins with high capacity and well-dispersed properties, which benefits the preparation of formulations employed for immunization.

Efficient antigen delivery into immune cells is crucial for inducing robust antibody responses in animals [29]. For the conventional formulation with Freund’s adjuvants, antigens are randomly trapped in water-in-oil droplets, effectively prolonging antigen exposure and enhancing antibody induction through depot formation [30]. In contrast, proteins stabilized on the ND surface form like an antigen array, which presents a constant format for immune cell recognition. Here, we detected that the average diameter of venom-conjugated NDs was approximately 948 nm, a size range known to facilitate uptake by APC through endocytosis or phagocytosis [31,32]. In mice immunization experiments, antigen-specific antibody responses were detected from high and low dosages of ND/venom groups, and their titers were comparable with those from the corresponding dosage of FA/venom immunogens. These results indicated that the ND/venom and FA/venom formulations might share similar immunogenicity to induce specific antibody responses in mice.

Further analysis of venom-specific IgG subclasses revealed that both IgG1 and IgG2a antibodies were induced in immunized mice. However, significantly higher IgG1 levels compared to IgG2a were observed, indicating a dominant Th2 immune response. Such a Th2-biased response was evident in both ND/venom (IgG1/IgG2a ratio > 2) and FA/venom (IgG1/IgG2a ratio > 1) groups. This is consistent with other investigation findings that ND- and IFA-based formulations preferentially induced Th2-biased immunity in the mouse model [16,33]. It is well established that Th2 responses promote humoral immunity that stimulates B cell proliferation and antibody induction. In line with the observations, our study showed that ND-based formulation might possess immunological characteristics similar to FA/venom immunogen in its capacity to enhance antibody responses.

Neutralizing medically important toxins is critical for protecting snakebite victims from the toxic consequences of envenoming. For *Naja atra* snake species, sNTX is recognized as the primary element contributing to the lethality of cobra venom. Therefore, the induction of antibodies to neutralize the toxicity of sNTX is essential for effective antivenom development. In this study, we demonstrated that ND/venom immunization successfully elicited specific antibodies against native sNTX protein (Figure 3a), with titers comparable to those induced by the FA/venom immunizations. Additionally, our study results confirmed that mouse sera raised by either ND/venom or FA/venom strongly recognize peptide fragments of sNTX (Figure 3c) encompassing the residues of lys27, trp29, Asp31, and Arg33 that are implicated in binding to muscular nAChRs and mediating neurotoxicity [34]. Notably, our previous research has shown that peptide-specific antibodies targeting these functional residues are highly associated with the ability to neutralize venom toxicity. Thus, our study findings suggested that ND/venom immunization can direct the antibody response toward functionally critical regions of sNTX, essential for effective neutralization. In the in vivo challenge experiment, all ND/venom or FA/venom groups of mice survived, while control groups succumbed rapidly following venom injection. This outcome indicated that immunized mice developed high-titer, neutralizing antibodies capable of rapidly inactivating the potent and lethal venom components. Importantly, these results supported that ND-based formulation could present venom antigens in a native-like structure and preserve critical conformational epitopes essential for eliciting neutralizing antibodies.

To assess the potential of ND/venom immunogen for antivenom production, we evaluated its capability to induce neutralizing antibody responses in the rabbit hyperimmunization model. By comparison, immunization with FA/venom elicited higher venom- and sNTX-specific antibody titers by week 4, while ND/venom reached peak titers slightly later (by week 8) and at significantly lower levels. However, rabbit IgG raised by ND/venom hyperimmunization showed nearly two-fold higher neutralization potency compared to those from the FA/venom group. This observation demonstrated the distinction between antibody titers and neutralization efficacy. Indeed, a similar phenomenon has been reported in other investigations, where antivenom raised by the venom of *N. kaouthia*, a cobra species widespread across South and Southeast Asia, displays comparable immunoreactivity to different types of cobra neurotoxins but varies substantially in their neutralization effectiveness [35]. In this study, we demonstrated that rabbit sera from these two immunogens showed different recognition profiles against sNTX-derived peptides. Rabbit sera from FA/venom showed a broad reactivity to peptides located at central loop II, as well as regions spanning around N- or C-terminal domains of sNTX. In contrast, the ND/venom group showed a more focused recognition pattern restricted to peptides encompassing the toxicity sites on loop II of sNTX. Given that antibody-mediated neutralization is closely linked to epitope specificity, our study findings suggested that ND/venom may selectively direct the immune response toward functionally critical epitopes, potentially enhancing neutralization efficacy even with lower overall antibody titers.

In the context of large-scale antivenom production, the clinical relevance of reducing local lesions at the injection site is particularly important for large animals such as horses, which are commonly used as antibody producers. Conventional adjuvants like Freund’s are known to induce local tissue damage, inflammation, or granuloma formation, which may lead to animal welfare concerns and complicate long-term immunization schedules. In contrast, our findings showed that venom ND/venom formulation elicited strong and neutralizing antibodies without inducing visible lesions in mice and rabbits. This improved local tolerability supports the translational potential of nanodiamond-based adjuvants as a safer and more sustainable alternative for large animal immunization programs in novel antivenom predevelopment and manufacturing.

## Figures and Tables

**Table 1:** List of the specific antibody titers and the neutralization potency of rabbit sera against cobra venom.

Rabbit Antisera Group	Antibody Titers (Endpoint, Log_10_)		Neutralization Potency (3LD_50_ Cobra Venom)	
	Anti-Venom	Anti-sNTX	ED_50_ (mg) ^1^	P (mg/g) ^2^
ND/venom	5.14 ± 0.02	4.20 ± 0.01	2.31 (1.78–2.83)	5.76 (4.03–10.08)
FA/venom	5.93 ± 0.03	4.60 ± 0.02	5.56 (4.84–6.32)	5.76 (4.03–10.08)

^1^ D_50_ represents the median effective dose, indicating the amount of rabbit antibodies (mg) protecting half of the mice under the challenging dosage of 3LD_50_ venom. ^2^ P indicates the amount of rabbit antibodies (g) to completely neutralize the amount of 3LD_50_ cobra venom.

## Data Availability

Data are available from the authors upon reasonable requests.

## Supplementary Materials

The following supporting information can be downloaded at: https://www.mdpi.com/article/10.3390/nano15191518/s1, Table S1: List of synthetic peptides derived from sNTX for immune recognition of serum antibodies.

## Abbreviations

The following abbreviations are used in this manuscript:

NDs	Nanodiamonds
CFA	Complete Freund’s adjuvant
IFA	Incomplete Freund’s adjuvant
APCs	Antigen-presenting cells
sNTX	Short-chain neurotoxin
nAChRs	Nicotinic acetylcholine receptors
LD_50_	Median lethal dose
DLS	Dynamic light scattering
ELISA	Enzyme-linked immunosorbent assays
