# Supplementary material for: Evaluation of Nanodiamond-in-Oil Emulsion with Snake Venom to Enhance Potent Antibody Induction in Mice and Rabbits
Source: Nanomaterials (Basel). 2025 Oct 4;15(19):1518. doi: 10.3390/nano15191518 (PMC12525691; doi:10.3390/nano15191518)
Supplement: Supplementary file 1 [file nanomaterials-15-01518-s001.zip › nanomaterials-3847214-supplementary.pdf]

Article

Evaluation of Nanodiamond-in-Oil Emulsion with Snake Venom to Enhance Potent Antibody Induction in Mice and Rabbits

Min-Han Lin [1], Long-Jyun Su [2], Hsin-Hung Lin [2], Liang-Yu Chen [2], Asmaul Husna [3] and Wang-Chou Sung [1,*]

Table S1. List of synthetic peptides derived from sNTX for immune recognition of serum antibodies.

Peptides [a]	Sequence
S1-15	LECHNQQSSQTPTTT
S4-18	HNQQSSQTPTTTGCS
S11-25	TPTTTGCSGGETNCY
S16-30	GCSGGETNCYKKRWR
S21-35	ETNCYKKRWRDHRGY
S26-40	KKRWRDHRGYRTERG
S31-45	DHRGYRTERGCGCPS
S36-50	RTERGCGCPSVKNGI
S39-53	RGCGCPSVKNGIEIN
S45-59	SVKNGIEINCCTTDR
S48-62	NGIEINCCTTDRCNN

Note. a. the number indicates the amino acid locations from mature sequence of sNTX (accession no. P60770, UniProt).
